# Theme and variations: evolutionary diversification of the HET-s functional amyloid motif

**DOI:** 10.1038/srep12494

**Published:** 2015-07-29

**Authors:** Asen Daskalov, Witold Dyrka, Sven J. Saupe

**Affiliations:** 1 Non-self recognition in fungi, Institut de Biochimie et de Génétique Cellulaire, UMR 5095, CNRS - Université de Bordeaux, 1 rue Camille Saint Saens, 33077 Bordeaux cedex, France; 2 Team MAGNOME, INRIA - Université Bordeaux – CNRS, 33405 Talence, France; 3 Department of Biomedical Engineering, Faculty of Fundamental Problems of Technology, Wroclaw University of Technology, 50-370 Wroclaw, Poland

## Abstract

In mammals and fungi, Nod-like receptors (NLR) activate downstream cell death execution proteins by a prion-like mechanism. In *Podospora anserina*, the NWD2 NLR activates the HET-S Helo-domain pore-forming protein by converting its prion-forming domain into a characteristic β-solenoid amyloid fold. The amyloid forming region of HET-S/s comprises two repetitions of a 21 amino acid motif. Herein, we systematically analyze the sequences of C-terminal regions of fungal HeLo and HeLo-like domain proteins to identify HET-s-related amyloid motifs (HRAM). We now identify four novel HRAM subfamilies in addition to the canonical HET-S/s subfamily. These novel motifs share the pseudo-repeat structure of HET-S/s and a specific pattern of distribution of hydrophobic and polar residues. Sequence co-variance analyses predict parallel in-register β-stacking of the two repeats and residue-residue interactions compatible with the β-solenoid fold. As described for HET-S, most genes encoding the HeLo proteins are adjacent to genes encoding NLRs also displaying HRAMs. The motifs of the NLRs are similar to those of their cognate HeLo-domain protein, indicating concerted evolution between repeats. This study shows that HET-s-related amyloid motifs are more common than anticipated and that they have diversified into discrete subfamilies that apparently share a common overall fold.

Amyloids are self-templating protein polymers assembled by stacking of β-strands^1^. Amyloid formation is often associated with disease, in particular neurodegenerative age-related pathologies such as Alzheimer’s or Parkinson’s diseases or other conditions like type 2 diabetes^2^,^3^. Amyloid formation has also been linked to cancer with a proposed role of p53 amyloids in tumorogenesis^4^. Yet, amyloids also fulfill a range of different functions inside and outside cells^5^,^6^,^7^. Such so-called functional amyloids are involved in hormone and toxin storage and release, formation of cell surface structures in microorganisms and scaffolding of pigment synthesis. A role in the regulation of cell states is also described in yeast, where prion amyloids behave as epigenetic regulatory switches^8^.

Recently, it has also been reported that functional amyloids play a role in cell fate controlling signaling cascades both in mammals and fungi. In mammals, the RIP1 and RIP3 kinases which control the necroptosis cell death pathway form an amyloid-like complex assembled via short conserved motifs termed RHIM (for RIP homotypic interaction motifs)^9^. In the fungus *Podospora anserina*, a prion amyloid motif is involved in the activation of the HET-S cell death inducing protein by a Nod-like receptor termed NWD2^10^,^11^. HET-S display two distinct domains, the N-terminal cell death execution domain termed HeLo and the C-terminal regulatory prion forming domain (PFD)^12^,^13^,^14^. The 72 amino acid long PFD is natively unfolded in the soluble form of the protein. The PFD adopts a β-solenoid fold with two rungs of β-strands per monomer each composed of an imperfect repeat of a 21 amino acid motif (Fig. 1A,B)^15^,^16^,^17^. Structural and functional studies suggest that HET-S homologs from other fungal species also adopt a related β-solenoid fold^18^,^19^, (Fig. 1B). Cell death is triggered when the PFD region of HET-S is converted to the β-solenoid fold. This in turn induces refolding of the HET-S HeLo domain which exposes a N-terminal hydrophobic α-helix causing HET-S to behave as a pore-forming toxin^12^. Upon transconformation, HET-S relocates from the cytoplasm to the cell membrane where it exerts toxicity^20^. Conversion of the PFD region can also be achieved by a protein termed NWD2 which is encoded by the gene immediately adjacent to *het-S* in the genome^10^,^11^. NWD2 encodes a Nod-like receptor with a NACHT and WD-domain and an N-terminal region homologous to the HET-S/s motif (Fig. 1B). Upon ligand-binding to NWD2, these N-terminal extensions adopt the β-solenoid fold and template HET-S to adopt the amyloid fold and trigger toxicity. NWD2/HET-S is one of several examples in which activation of a cell death and host defense cascade relies on a prion-like polymerization mechanism^21^,^22^. Two other putative functional amyloid motifs associated with genomically clustered NLR and effector proteins were identified in fungi and designated σ and PP^11^. The HET-s motif differs from these additional motifs by its pseudo-repeated structure, with two copies of the motif found on the effector protein and a single copy of the motif found in the NLR. It has been reported that the RHIM controlling mammalian necroptosis and the HET-S motifs might be evolutionarily related^23^.

Herein, we conduct a bioinformatic analysis of a dataset of 579 fungal HeLo domain proteins and analyzed C-terminal regions for the presence of putative amyloid motifs. In addition to the σ and PP motifs which had been described previously and are present as a single copy in the HeLo domain protein, we have identified novel HET-s-related motifs. Like the canonical HET-s-motif, these motifs are organized as imperfect repeat pairs in the C-terminal region of the HeLo domain proteins. We collectively analyze these motifs and find that they correspond to four new subfamilies of HET-s-related motifs.

## Results

### Identification of duplicated amyloid motifs in HeLo, HeLo-like and sesA domain proteins

The HeLo domain is a cell death execution pore-forming domain originally identified in the HET-S protein and inducing a form of programmed cell death in *Podospora anserina*^12^,^13^. Two additional domains termed HeLo-like and sesA share a remote homology with the HeLo domain and are found in the same type of domain associations as HeLo^11^,^24^. In particular, HeLo, HeLo-like and sesA domains often occur as N-terminal effector domains of NLRs or alternatively display C-terminal prion forming motifs. In the latter case, and as described for HET-S and NWD2, these proteins are encoded by genes that reside adjacent to genes encoding NLRs sharing the same motif. In order to systematically explore the C-terminal extension of these HeLo, HeLo-like and sesA domain proteins, we screened fungal genomes for proteins homologous to prototypical HeLo, HeLo-like and sesA proteins chosen as queries (respectively *P. anserina* HET-S, *P. anserina* Pa_3_9900, *Chaetomium globosum* CHGG_01412 and *Nectria haematococca* sesA) in blastp searches with a cutoff at 10^−2^. This allowed the recovery of 1420 sequences. To eliminate NLR associated HeLo-related domains (which occur in large proteins of >800 amino acids in length), only sequences of less than 310 amino acids in length were retained. Doing so, a dataset of 579 fungal HeLo-related proteins was obtained. The regions corresponding to the last 80 C-terminal amino acids were extracted from each sequence and analyzed for presence of motifs using MEME. Three main classes of motifs were identified. Two of these motifs correspond to the previously defined σ and PP signatures 76 and 98 hits respectively^11^. The σ and PP groups are not analyzed further herein. The third and most frequent motif is related to the canonical HET-s-amyloid motif and was generally (but not always) repeated twice in the selected 80 aa C-terminal region. There is perfect conservation of a glycine residue (located in a β-arch position in HET-S/s, position 19) and conservation of a pattern of hydrophobic residues (which form the core in HET-S/s) at positions 5, 8, 10, 16 and 18^18^,^25^. We focused our analysis on this group of sequences and attempted to isolate the first and second repeat motif (r1 and r2) in each sequence. To that end, the region matching the MEME motif was extracted, mapped back to the original sequence and local alignments were performed for each sequence to find the second motif. The procedure allowed for the identification of 155 sequences with two repeats, that is 310 repeat motifs (file S1).

**Figure 1:**
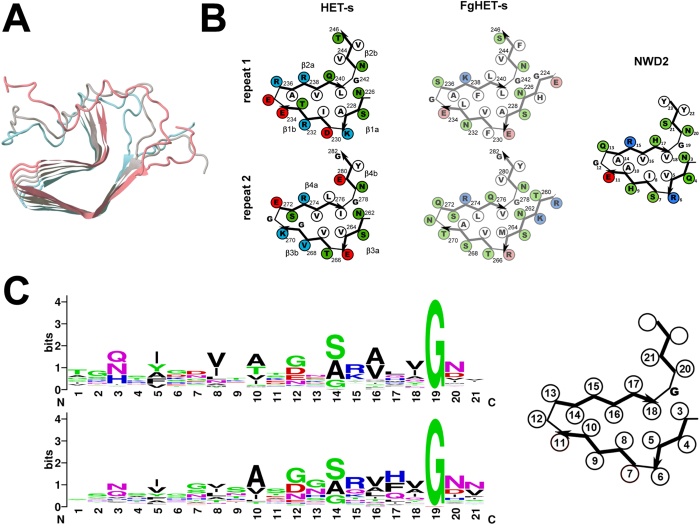
Structure of the reference HET-s-motif and consensus sequence of the ensemble of HET-s-related motifs. (**A**) Structure of the HET-s prion forming domain (after PDB 2KJ3), three monomers are depicted each with a different colour. (**B**) Structure diagram of the first and second repeat of the prion forming domain region of *Podospora anserina* HET-s and *Fusarium graminearum* (FgHET-s) as well as a structure model for the HET-s-motif of NWD2. Diagrams are after PDB 2KJ3 for HET-s and structural models based on solid state NMR and hydrogen exchange data for FgHET-s and solid state NMR for NWD2. (**C**) Sequence logo giving the consensus sequence for the ensemble of 310 HET-s-related motifs (upper diagram) and for the ensemble of 98 r0 HET-s-related motifs on associated NLRs. Numbering is according to the HET-s-motif model recalled on the right.

The local alignments required introduction of gaps in the first repeat in a small fraction of the sequences. The two repeats are connected by a region of variable length. 154 out of 155 sequences had a loop of 6 to 18 amino acids in length while a very short interconnecting region of only 2 amino acids was found in just one sequence. Loop primary sequence was not conserved but was usually rich in G residues (average G content in loop regions is 14.2% compared to 7.2% in all of Uniprot). The second repeat was not located at the extreme C-terminus of the sequence but was followed by a short segment often containing aromatic residues and diglycines. In HET-s, this region corresponds to a functionally critical semi-flexible loop folding back onto the core^16^,^25^. Of the 155 sequences identified with a double repeat motif only 38 matched the Pfam signature for the HET-s (218–289) prion forming domain (PF11558), indicating the existence of numerous additional divergent repeated motifs associated to HeLo-related domains in fungi. Based, on the conservation of the hydrophobic/polar residue pattern and of β-arch forming positions, the sequence of these additional motifs appears compatible with the β-solenoid fold (Fig. 1C).

### Repeat motifs fall into subfamilies and segregate by repeat order

**Figure 2:**
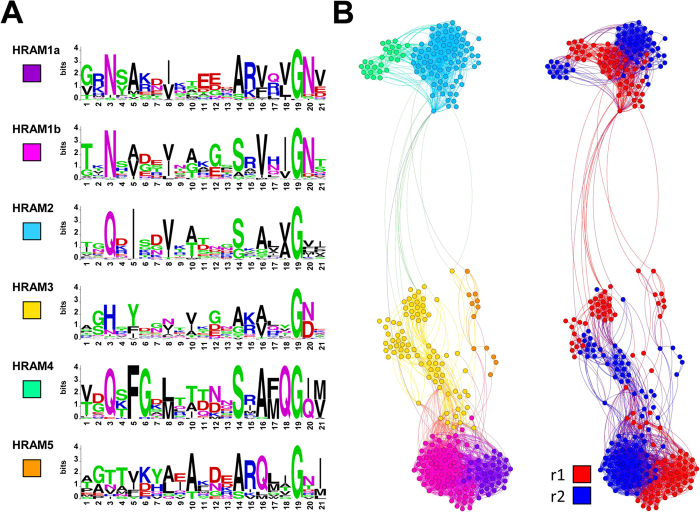
Clustering of HET-s-related motifs from HeLo-domain proteins. (**A**) Sequence logos defining subfamilies of HET-s-related motifs from fungal HeLo-domain proteins at PAM110. (**B**) Graphical representation of clustering of the individual HET-s-related motifs in the subfamilies defined in A. (left constellation) and classified by repeat order (right constellation). The figure shows the six most numerous groups (including 300 out of 310 motifs) that formed a single inter-connected supercluster.

In order to determine if the newly identified motifs fall into discrete subfamilies or form a continuum of variants, we compared the 310 repeat sequences to determine their clustering. Clustering graphs based on distances calculated by the JTT method and a cutoff of PAM 110 or PAM130 were generated and sequence logos for the main groups were obtained. At a low cutoff value, 6 groups were identified (Fig. 2A). The different families were designated HRAM1 to HRAM5 (Het-s-Related Amyloid Motif). Repeat order was attributed to the sequences and it turns out that HRAM1a and HRAM1b are related respectively to repeats r1 and r2 of the canonical HET-s-type sequences^25^. The sequence logos for these groups are essentially identical to the r1 and r2 consensus sequences previously generated for close HET-s homologs^25^. In all groups, there is conservation of the G residue in position 19 and in general hydrophobic residues are found in 5, 8, 10, 16 and 18, the positions that make up the hydrophobic core in HET-s^17^. At position 10 as already observed for HET-s-related sequences (HRAM1a and b), a T residues is also sometimes found. In all motifs there is a preference for a positively charged R (or K) residue in 15. In contrast, the motifs differ in position 3, which is N in canonical HET-s (HRAM1a and b) but Q in HRAM2 and H in HRAM3. HRAM4 and 5 are also characterized by presence of a Q in position 18 and 16 respectively, never seen in the canonical HET-s motif and predicted to be located inside the core. In all subfamilies, sequences cluster by repeat order, in other terms, motifs segregate by HRAM group and then within a group by repeat order (Fig. 2B).

When the same procedure is performed at cutoff PAM130, sequences resolve in just three supergroups (Fig. S1). The first corresponds to sequences with a Q in position 3 and merges HRAM2 and 5. A second group corresponds to HRAM3. A third supergroup merges HRAM1a and b and HRAM 5. Eleven sequences which belonged to HRAM3 at the PAM110 cutoff merged with HRAM1 at the PAM130 cutoff suggesting a somewhat blurred delimitation between HRAM1 and 3. In each of the three supergroups, there is again subclustering by repeat order (Fig. S1B).

We conclude from this analysis that HET-s-related amyloid motifs can be differentiated into five groups that display variation around the typical HET-s-motif. In each of the groups, there is clustering of sequences by repeat order. Importantly, clustering is first by group and then by repeat order, suggesting concerted evolution between the two repeats in each group.

We have analyzed the species distribution of the different groups of HET-s-related motifs. Our data set of 155 HeLo-related proteins with HRAM motifs, corresponded to 79 different species. Of those, 29 species encoded at least two HeLo-related proteins with HRAM motifs and 17 species have motifs belonging to different groups (Table 1). Thus, in numerous species HET-s-related motifs from different groups co-exist. For instance in *Podospora*, in addition to a HET-s (HRAM1 group), a Helo-like domain protein with a HRAM4 motif is found.

### Analysis of intra and inter-motif sequence co-variance predicts structure

In HET-s, there is formation of three inter-repeat salt bridges (at position 6, 11 and 13) (Fig. 1B) and these salt bridges were found to contribute to fibril formation, stability and prion function^16^,^17^,^25^,^26^. It was observed previously that in the sequences of close HET-s-homologs, favorable charge interactions between the two repeats are predicted^18^. We have thus analyzed the 155 motif pairs for charge correlations between repeats. We find that there is significant anti-correlation between charges (that is charge complementarity) at equivalent positions between the two repeats at positions 4, 6, 7, 11, 12 and 13, namely all positions predicted to be located outside of the core (with exception of position 15 in which there is overall conservation of a positive charge) (Fig. S2). This observation is consistent with the hypothesis stating that repeat r1 and repeat r2 of each sequence undergo in register β-stacking and that salt bridges stabilize the fold as described in HET-s. Charge correlation was also examined within each repeat and significant anti-correlation was found between position 17 and 21 within repeats both for r1 and r2, an observation again consistent with the spatial proximity of residue 17 and 21 predicted by the HET-s β-solenoid structure (Fig. S2).

**Figure 3:**
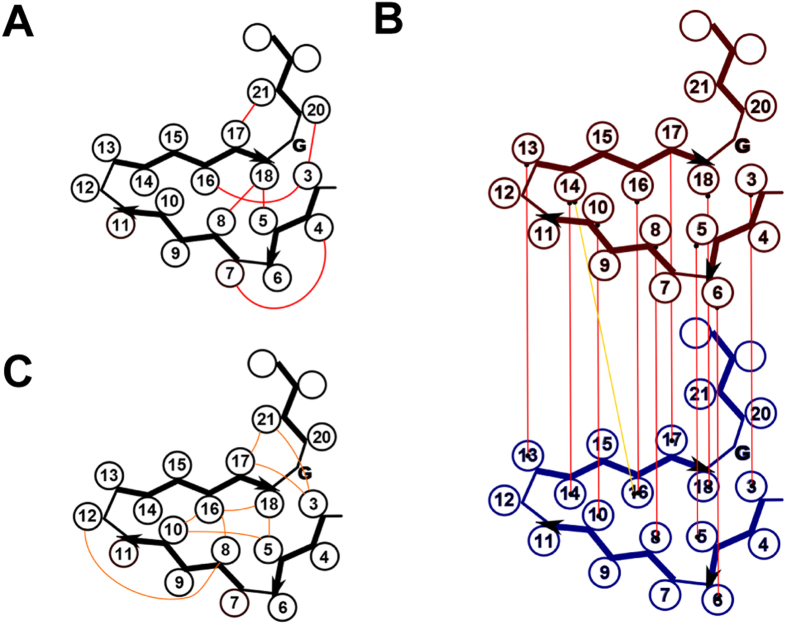
Sequence co-variance in HET-s related motifs. (**A**) HET-s-related motif variation was analyzed with Gremlin for intra-repeat sequence correlation and significant (*i, j*) co-variance pairs were represented by red lines on the structural model of the elementary HET-s-repeat model. (**B**) Inter-repeat variation was analyzed with Gremlin and significant (*i, j*) co-variance pairs were represented on the structural model of the elementary HET-s-repeats, in- register correlations are given red, out-of-register correlations in orange. **(C)** HET-s-related motif of associated NLRs were analyzed for intra-repeat sequence correlation with Gremlin, significant (*i, j*) co-variance pairs were represented by orange lines on the structural model of the elementary HET-s-repeat model.

Analysis of evolutionary co-variation has been successfully used to predict residue-residue contacts within proteins or protein complexes and to infer structural constraints for structure prediction^27^. This approach has been applied essentially for soluble and membrane proteins but a recent study has shown that the method is also applicable to functional amyloids. This approach was used to infer structural information from the natural variation in the bacterial CsgA protein forming the curli amyloid fibrils^28^. We thus decided to analyze our HET-s-related motif data set for sequence co-variation in order to determine if predicted potential residue-residue contacts are consistent with conservation of the β-solenoid fold. We employed the Gremlin algorithm to predict residue-residue co-variation in our data of HET-s-related motifs^27^. Cut-offs were set at scaled score >1.25 and probability >0.99. Six residue-residue interactions were predicted for the following (i;j) pairs : (8;18), (3;16), (5,18), (4;7), (17;21) and (3;20). These predicted constraints are compatible with the HET-s structure and match experimental constraints obtained by ssNMR^16^,^17^. In particular, residue-residue contacts between hydrophobic residues in positions 5 and 18, and 8 and 18 are accurately predicted as well as proximity of position 3 and 20 and 17 and 21 (Fig. 3A). Co-variance is also detected between positions 4 and 7, which cannot *a priori* be accounted for by a direct intramolecular residue-residue contact. Co-variance between positions 3 and 16 could be an indirect effect of variation at position 18.

The same analysis was repeated using the r1 and r2 repeats of each sequence from our data set, in order to detect co-variation between repeats. Cut-offs were set at scaled score >1.25 and probability >0.85. In addition to the intra-repeat pairings described above, 11 residue-residue co-variations between repeats were predicted. Of those ten corresponded to in-register pairing of the two repeats at positions 3, 5, 6, 8, 10, 13, 14, 16, 17 and 18 and one pairing between position 14 of repeat 1 and position 16 of repeat 2. These constraints are consistent with a parallel in-register pairing of the two repeats as seen in the HET-s β-solenoid structure (Fig. 3B).

Charge correlation between repeats, as well as co-variance analyses are consistent with the prediction that HET-s-related sequences adopt a structure similar to the canonical HET-s β-solenoid fold. These results illustrate that analysis of evolutionary co-variance in a functional amyloid can accurately predict structural constraints.

### Co-evolution between motifs in HeLo domain proteins and their associated NLR

In *Podospora anserina*, NWD2 is encoded by the gene immediately adjacent to the HET-S-encoding gene. More generally, genes encoding HeLo-domain proteins with a HET-s-type prion forming domain also show genomic clustering with the gene encoding their cognate regulatory NLR protein in different species^10^. To determine if this type of genomic clustering is also detected for the HET-s-related motifs identified herein, we have systematically analyzed the sequence of the gene immediately upstream or downstream of the gene encoding the 155 HeLo-related proteins in our data set. For 98 of those, NLR encoding genes were found in the adjacent position. The corresponding NLRs displayed a single copy of a HET-s-related motif at their N-terminus. It thus appears that genomic clustering of the genes encoding HeLo-related protein and their regulatory NLR is a common but not systematic feature.

When analyzed collectively, these NLR motifs resemble the general HET-s-motif in particular with a conserved pattern of hydrophobic residues in positions 5, 8, 10, 16 and 18 (Fig. 1C). The NLR motifs are however characterized by a lower occurrence of charged residues. When the amino acid content of the motifs is analyzed, one notes a marked decrease in D, E and K and in contrast a higher content in S and H (Fig. S3). This observation could be explained by the fact that homotypic stacking of charged residues of the r0 motifs of the NLRs produces repulsive interactions, whereas charge complementarity can occur during heterotypic stacking of r1 and r2 motifs. Thus surface charged residues might be replaced by non-charged polar residues (such as S and H) at external positions in r0 motifs. Co-variance was also analyzed in the data set of NLR r0 motifs. Cut-offs were set at scaled score >1.25 and probability >0.85 as above. Nine significant co-variances were detected at that cut-off. Most were consistent with the β-solenoid fold (Fig. 3C). A relationship between position 8 and 12 was also detected which cannot be interpreted as a direct interaction in a HET-s-like β-solenoid fold. The coupling matrix for this specific residue pair indicates that position 12 varies with the type of hydrophobic residue in position 8. I and L in position 8 are correlated with G in the arch position 12 while V is correlated with D. It might be that a relationship between the geometry of the hydrophobic core and the β-arch position (rather than a direct contact) can explain this evolutionary coupling and co-variance.

**Figure 4:**
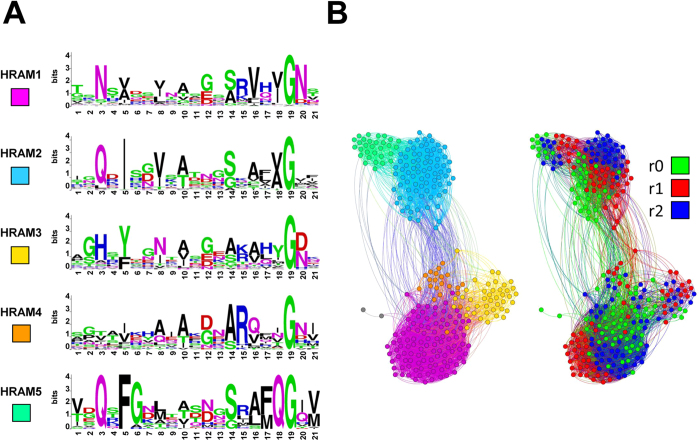
Clustering of HET-s-related motifs from HeLo-domain proteins and their associated NLRs. (**A**) Sequence logos defining subfamilies of HET-s-related motifs from fungal HeLo-domain proteins at PAM130. (**B**) Graphical representation of clustering of the individual HET-s-related motifs in the subfamilies defined in A. (left constellation) and classified by repeat order (right constellation). The logos were generated for the five most numerous groups (including 277 out of 285 motifs). Two other motifs connected with the main supercluster are shown in grey in B.

Next, we have analyzed the clustering of r0 motifs and found that they also fall into discrete subfamilies like the r1 and r2 motifs (Fig. S4). When clustered with the r1 and r2 motifs, r0 most generally belong to the same group as their associated r1/r2 (Fig. 4, Table 2). In some subgroups in particular HRAM1 (canonical HET-s) and HRAM4, r0 motifs tend to cluster with r2 (*ie* are more similar to r2 than to r1) but apparently in HRAM2 the opposite trend occurs. The fact that r0 motifs are more closely related to r2 than to r1 was noted before for canonical HET-s-motifs^10^. At any rate, the fact that r0 motifs resemble the r1/r2 motifs of their cognate NLR indicates co-evolution between the HeLo protein and the corresponding NLR, and thus suggests that motifs have diverged in a concerted manner. The frequent occurrence of genomically clustered NLRs and HeLo-domain proteins sharing common HET-s-related motifs and their concerted evolution supports a conserved role for these motifs in signal transduction in the fungal phylum, as described for the NWD2/HET-S pair^10^.

## Discussion

In the fungus *P. anserina*, the NWD2 protein signals activation of the HET-S HeLo-domain cell death execution protein by a mechanism of amyloid templating. The amyloid structure of HET-s is known and corresponds to a characteristic β-solenoid fold^17^. Herein, we have systematically analyzed the C-terminal regions of fungal HeLo-domain proteins to identify potential HET-s-related amyloid motifs in fungal genomes to study the molecular evolution of a functional amyloid motif in a context in which structural information is at hand.

### Expanding the HET-s family: theme and variations

Here, in addition to the canonical HET-s amyloid motif, four other HET-s related motifs are identified, indicating that significance of this type of motif is wider than previously anticipated. Roughly, half of the HeLo-related proteins of <310 amino acid in length, bear a HET-s-related motif with only approximately 25% of those responding to the HET-s (218–289) Pfam signature. PP and σ motifs are also frequently found but the HRAM family remains the most common category of HeLo-associated amyloid motifs. The HET-s-related motifs occur as two repeats separated by a poorly conserved intervening region which in HET-s corresponds to a flexible loop. In many sequences also, a short region containing G-residues and aromatics occurs C-terminally to the second motif. In HET-s, this region forms a functionally relevant semi-flexible loop making up a hydrophobic pocket that extends the core^16^,^25^.

Conservation of key features between the HET-S/s PFD and these motifs described herein can be taken as an indication that these other motifs adopt a fold that is related to the β-solenoid fold. Apparently, conservation of this fold requires only loose sequence constraints illustrated here by the general sequence logo of the HET-s-motifs (Fig. 1C). Key features are the pattern of hydrophobic/polar residues and the β-arch forming G residue at the end of the motif. Mutational studies also suggest that the HET-s fold is robust and accommodates various sequence alterations^25^,^26^. Very few point mutations in the HET-s-motifs were able to affect the overall β-solenoid fold. It appears that the β-solenoid fold can accommodate a large number of sequence variations as long as the pattern of hydrophobic to polar residues is maintained and that β-arch formation can occur. Many of the HET-s related sequences identified herein show less than 10–15% identity in primary sequence to HET-s r1 and r2. In general terms and considering that all motifs conform to the β-solenoid fold, the common features of the sequences can be described as four stretches of β-strands with alternating polar and hydrophobic residues interrupted by three β-arch forming regions. Interruption can be obtained by two or three consecutive polar residues (arch 1 and 2, respectively) or a single glycine (arch 3). Finally, large polar residues in positions 3 and 20 might allow sealing of the core. Variation around this theme occurs on the polar residues in positions 3 and 20 and the presence or absence of Q polar residues in the third strand in positions 16, 17 or 18. Co-variance analysis predicts an interaction between positions 17 and 21, suggesting that the roughly perpendicular orientation of strands 3 and 4 seen in the HET-s structure, is a common feature of the different motifs. Examination of the co-variance matrix for these two positions suggests that this interaction can be achieved by two different means. Presence of a hydrophobic residue in position 17 is correlated with presence of a hydrophobic residue in position 21 and conversely, presence of a polar residue in position 17 predicts a polar residue in position 21. This can for instance be spotted in the sequence logos in Fig. 2A. Thus interaction between strand 3 and 4 in positions 17 and 21 may result from a hydrophobic interaction or interactions between polar residues for instance by charge complementarity. In HET-s, three salt bridges per monomer stabilize the β-solenoid fold at positions 6, 11 and 13. In the other motifs, complementary charge interactions also occur but not necessarily at the same positions (anti-correlation between charges was detected at positions 4, 6, 7, 11, 12 and 13, Fig. S2). In addition, the relationship between charge and repeat order is variable. One common feature of the sequences, the conservation of the positive charge in position 15, remains unexplained.

Given the tolerance of the β-solenoid fold to sequence variation, it is interesting to note that the divergence of the motif does not correspond to a continuous drift. Rather than forming a continuum of variants, the identified motifs fall into discrete classes. This observation indicates that evolution has led to a diversification of this amyloid motif into subfamilies. In addition, clustering first by subfamily and then by repeat order within each class indicates that the two repeats in each sequence evolve in a concerted matter. The same is true also for the r0 motifs found in associated NLRs. The formation of discrete subclasses of motifs might indicate a functional diversification of the motif to allow formation of discrete independent signaling pathways. The fact that additional amyloid-forming motifs have been identified in fungi (σ and PP) often co-existing within the same species suggests that a biological need for several distinct signaling domains exists. What we explicitly suggest is that based on the same overall fold, HET-s motifs have diversified into distinct classes that no longer cross-seed. Obviously, functional studies are now required to validate the prediction that these other motif classes also have a β-solenoid fold and to determine to which extent cross-seeding does or does not occur between different motif subfamilies.

An analogy emerges here between the HET-s-related motifs and the DD (death domain) superfamily. The DD superfamily mediates activation of apoptotic and inflammatory complexes in mammals. These domains occur, among other architectures, as N-terminal domains of NLRs^29^. Thus death domains and HET-s motifs play similar functional role in cell fate signaling pathways and have even been found to be functionally interchangeable in that role. The HET-s prion forming domain was found to be able to substitute for Pyrin death domains in NLRP3/ASC signaling in mammalian cells^21^. DD and HET-s-like amyloid motifs function similarly by formation of higher-order prion-like complexes but achieve this by radically different structural means, namely association between α-helical folded death domains or amyloid templating in the case of HET-s motifs^10^,^21^,^22^. The DD superfamily is subdivided into several subfamilies (DD, CARD, DED, PYD) that share an overall fold but undergo essentially homotypic interactions. In this family also, it was proposed that functional diversification into discrete subfamilies represents a complexification of the role of this domain in various parallel signal transduction cascades^29^.

### Evolution predicts structure in functional amyloids

Analysis of co-variance in protein sequences has become a popular tool for predicting structural constraints or identifying interacting residues in protein complexes^27^. Until very recently, the method has not been applied to functional amyloids^28^. A previous pioneering report on CsgA and the present work indicate that analysis of co-variance can be a useful tool to predict residue-residue proximity in functional amyloids. In the case of CsgA, residue-residue contacts predicted by co-variance analysis were consistent with previous solid-state NMR, electron microscopy and fiber diffraction studies. Applied to our dataset of HET-s-related motifs, co-variance analysis was able to accurately predict expected intra-repeat and inter-repeat associations. While, the vast majority of couplings identified in these approaches are apparently structurally consistent, it should be noted, that not all detected instances of co-variance translate to an expected residue-residue contact or proximity. This is for instance illustrated in the co-variance analysis of the r0 motifs of NLR which identifies a co-variance between position 8 and 12 which cannot be explained by contact or proximity in the β-solenoid fold (Fig. 3C). Yet the work on CsgA and the present study might be taken as an incentive to apply co-variance studies to the structural and functional characterization of amyloids, especially considering that their structural dissection is extremely challenging. Of course, this approach is only applicable to evolved (functional) amyloids and not to disease-related amyloids.

This study illustrates the modalities and constraints that determine functional amyloid evolution. It comes may be as no surprise, that these modalities appear to be essentially similar to those governing evolution of every other native soluble or membrane protein fold. It has been stated that complexity of amyloid folds may fall somewhere between secondary and tertiary structure^5^, which may explain both the high tolerance of the HET-s-related motifs to variation but also their ability to diversify into discrete subfamilies.

## Methods

### Identification

Fungal homologues of prototypical HeLo (XP_001903320.1), HeLo-like (XP_001220633.1) and XP_001909800 and sesA (AAS80313.1) proteins were retrieved from the NCBI “nr” (as of 14/10/2014) and JGI (as of 1/11/2014) databases using blastp with e-value <10^−2^ 30. Out of them, 579 sequences, which were at most 310 amino acids (aa) long were retained and their C-terminal regions of 80 aa were extracted.

Preliminary analyses using MEME^31^ showed that HET-s-related motifs were the most common gap-less motifs in the dataset. Then, MEME was used to extract a single most frequent motif with at least 500 occurrences (any number of repetitions per sequence), and at least 17 aa long. The resulting 24 aa-long motif (500 instances) was trimmed to 17 residues (corresponding to positions between the conserved N and G in the classical HET-s motif^11^). Next, distances between instances of the MEME motif were calculated using fprotdist^32^ with the Jones-Taylor-Thornton (JTT) model^33^ from the EMBOSS package^34^. To purify the set from noise, only 273 instances which formed the largest inter-connected cluster at the PAM 100 distance were retained.

In order to make sure that the most comprehensive set of HET-s-related motifs is extracted, each C-terminal region with at least one MEME hit was searched for the second copy of the motif, a constitutive feature of the HET-s fold, using EMBOSS matcher (Waterman-Eggert algorithm^35^,^36^), with gap open and extend penalties 10 and 1, respectively. The match was accepted only if the longest alignment, out of best 10, had the score of at least 20 and the length of at least 15. After that, all C-terminals were searched for very close matches of the already found motifs using EMBOSS water (Smith-Waterman algorithm^37^), with the prohibitive gap cost and the score threshold of 40. The parameters of local alignments were chosen to obtain virtually perfect consistency with the MEME hits for sequences where the latter found two instances of the motif. Two cases with conflicting positions of the motif were manually resolved, and one false positive sequence had to be removed. Finally, 155 sequences with a double occurrence of the HET-s-related motif were found. The set of 310 motifs was manually re-aligned and extended by two amino acids at both sides.

In the next step, NLR encoding genes were searched immediately upstream or downstream of the genes encoding the 155 HeLo-related proteins with double occurrence of the HET-s-related motif. For 98 cases in which NLR genes were found in the adjacent position, single HET-s-related motifs were manually extracted and aligned. Three cases with an uncertain alignment were excluded from further analysis. In result, the set of triple occurrences of HET-s-related motif consisted of 95 pairs of HeLo-related and NLR proteins (285 motifs).

### Analyses

Clustering graphs, such that motifs were represented by nodes, and distances below a given threshold (PAM 110 or PAM 130) represented by edges, were generated, and visualized using the Force Atlas method^38^ in gephi^39^. Distances between motifs were calculated using fprotdist and the JTT model. Modularity classes in the graph were computed using the Louvain method^40^ at resolution 1.0 with randomization, implemented in gephi. For each motif group (modularity class), a sequence logo was generated by weblogo 2.8^41^.

Correlations (Pearson’s r) of formal charges were calculated for pairs of positions such that mutual information exceeded 0.1 nats, using R packages Hmisc^42^ and infotheo^43^. Only correlations with p-values below 10^−3^ were considered as significant. Residue-residue co-variations were predicted by web-server Gremlin v2 (http://openseq.org/gremlin_faq.php) without MSA enrichment.

## Additional Information

**How to cite this article**: Daskalov, A. *et al*. Theme and variations: evolutionary diversification of the HET-s functional amyloid motif. *Sci. Rep*. **5**, 12494; doi: 10.1038/srep12494 (2015).

## Supplementary Material

Supplementary Information

Supplementary File S1

## Figures and Tables

**Table 1:** Species distribution of Het-s-related motifs.

species	HRAM1	HRAM2	HRAM3	HRAM4	HRAM5	total (nb of classes)
*Aaosphaeria arxii*	—	1	—	—	1	2 (2)
*Anthostoma avocetta*	1	—	2	—	—	2 (2)
*Apiospora montagnei*	1	1	—	2	—	4 (3)
*Aspergillus fumigatus*	—	2	—	—	—	2 (1)
*Cadophora*	—	2	—	—	—	2 (1)
*Cladonia grayi*	—	1	—	—	1	2 (2)
*Cladophialophora carrionii*	—	—	2	—	—	2 (1)
*Cochliobolus sativus*	—	1	1	—	—	2 (2)
*Colletotrichum gloeosporioides*	—	—	2	—	1	3 (2)
*Coniochaeta*	—	—	—	2	—	2 (1)
*Corynespora cassiicola*	—	2	—	—	—	2 (1)
*Curvularia lunata*	—	1	1	—	—	2 (2)
*Fusarium graminearum*	4	2	—	—	—	6 (2)
*Fusarium oxysporum*	22	3	—	—	—	25 (2)
*Fusarium pseudograminearum*	3	—	—	—	—	3 (1)
*Hypoxylon*	1	—	1	—	—	2 (2)
*Ilyonectria europaea*	1	—	—	—	1	2 (2)
*Melanconium sp*.	2	—	—	—	—	2 (1)
*Meliniomyces bicolor*	1	—	1	—	—	2 (2)
*Nectria haematococca*	3	1	—	—	—	4 (2)
*Pestalotiopsis fici*	—	—	—	2	1	3 (2)
*Podospora anserina*	1	—	—	1	—	2 (2)
*Polyplosphaeria fusca*	—	1	1	—	—	2 (2)
*Pseudogymnoascus pannorum*	—	—	6	—	—	6 (1)
*Sphaerobolus stellatus*	—	—	4	—	—	4 (1)
*Thielavia arenaria*	2	—	—	—	—	2 (1)
*Thielavia hyrcaniae*	2	—	—	3	—	5 (2)
*Thozetella*	1	—	1	—	—	2 (2)
*Westerdykella ornata*	—	1	1	—	—	2 (2)

**Table 2:** Clustering of r0, r1 and 2 sequences into main groups at PAM130 and number of cases when r0 sequences are in the same group as their cognate r1 or/and r2.

Group	r1	r2	r0	r0&r1&r2	r0&r1	r0&r2
HRAM1	31	36	36	30	30	34
HRAM2	28	28	25	25	25	25
HRAM3	16	14	12	11	12	11
HRAM4	10	10	10	10	10	10
HRAM5	7	7	7	6	6	6
total	92	95	90	82	83	86
