# Supplementary material for: Theme and variations: evolutionary diversification of the HET-s functional amyloid motif
Source: Sci Rep. 2015 Jul 29;5:12494. doi: 10.1038/srep12494 (PMC4518210; doi:10.1038/srep12494)
Supplement: Supplementary Information [file srep12494-s1.pdf]

Supplementary figures

Theme and variations, evolutionary diversification of the HET-s functional amyloid motif

Asen Daskalov[£], Witold Dyrka[£] and Sven J.Saupe

Non-self recognition in Fungi

Institut de Biochimie et de Génétique Cellulaire

UMR 5095, CNRS - Université de Bordeaux,

1 rue Camille Saint Saens

33077 Bordeaux cedex France

[£]equal contributors

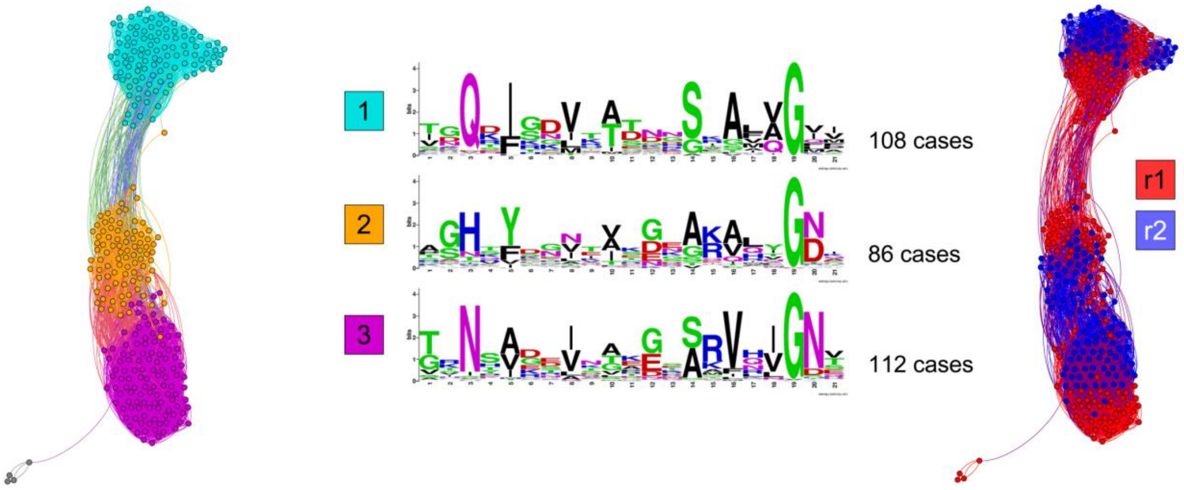

Figure S1. Clustering of HET-s-related motifs from HeLo-domain proteins at PAM130

A. Sequence logos defining subfamilies of HET-s-related motifs from fungal HeLo-domain proteins at PAM130 **B.** Graphical representation of clustering of the individual HET-s-related motifs in the subfamilies defined in A. (left constellation) and classified by repeat order (right constellation).

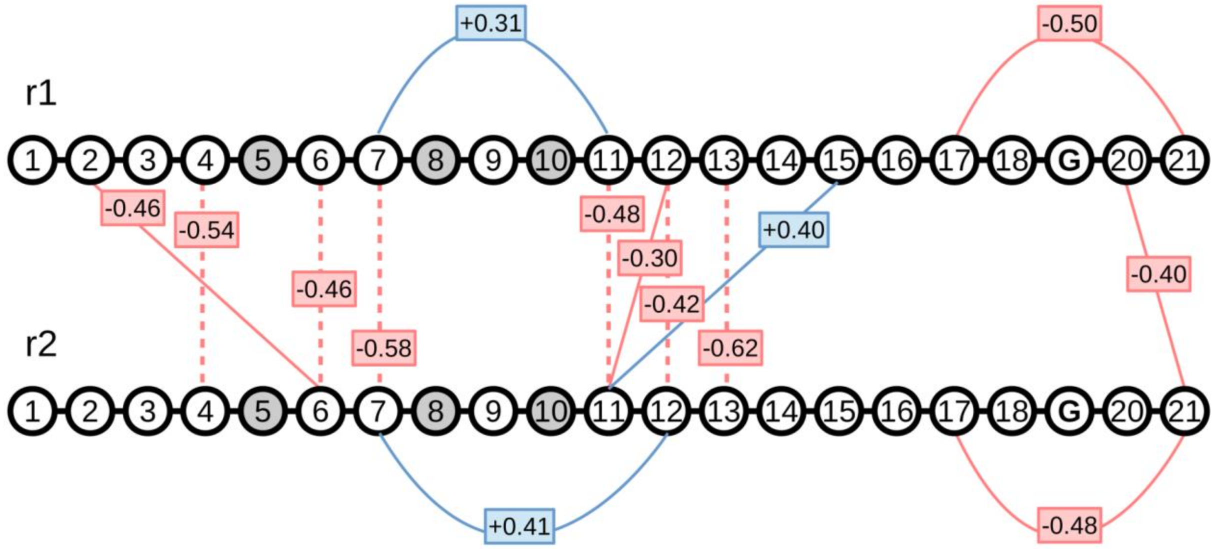

Figure S2. Intra and inter-repeat charge correlation in HET-s-related motifs from fungal HeLo-domain proteins

Repeats r1 and r2 of the HET-s-related motifs are represented linearly. Significantly correlated pairs of positions are connected by lines annotated with the Pearson's r coefficient (see Methods). Negative and positive correlations are indicated by red and blue colours, respectively. Inter-repeat in-register correlations are distinguished by dashed lines. Positions of the conserved hydrophobic residues are marked in gray.

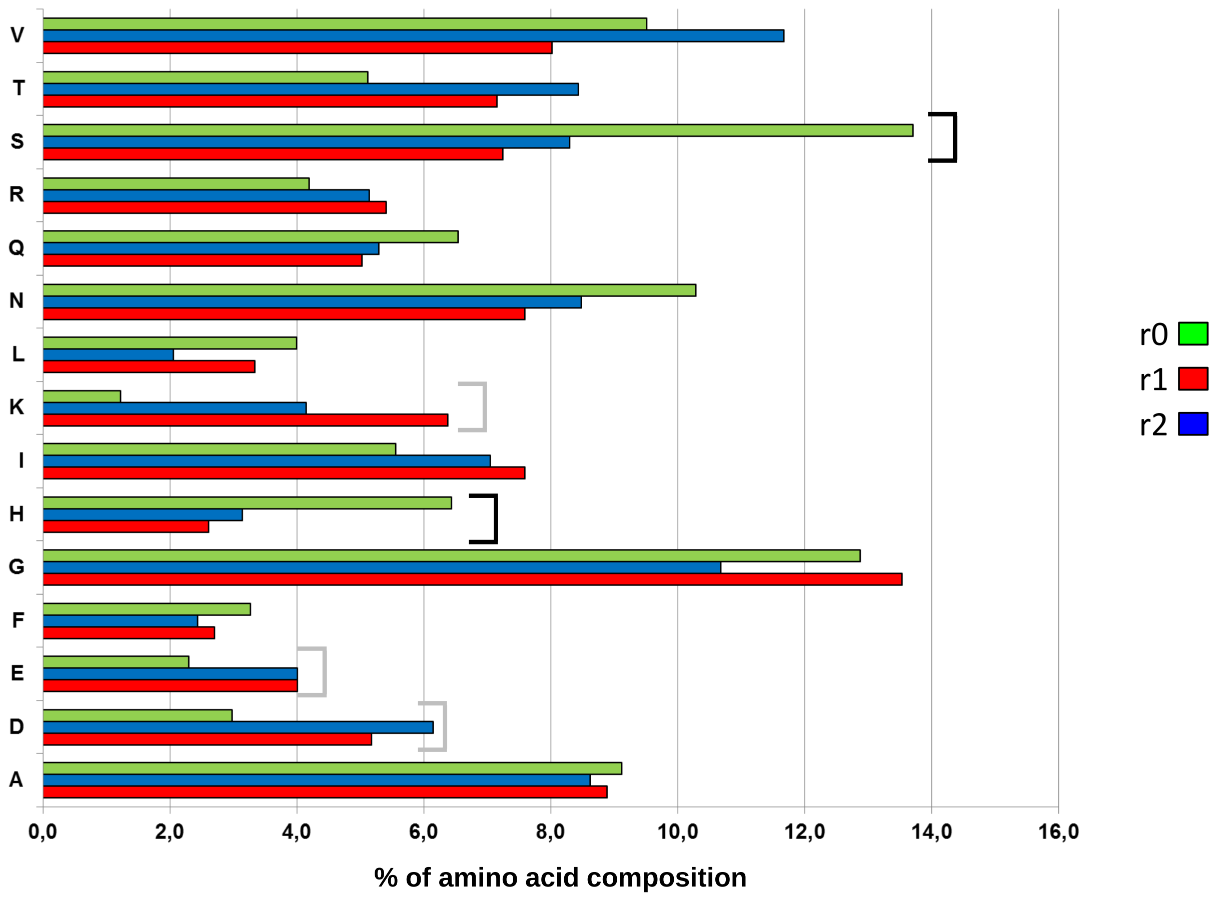

Figure S3. Comparison of the average amino acid composition in HRAM r0 motifs and r1/r2 motifs

For the listed amino acids, the ratio of the r0-motif amino acid composition over the r1/r2 composition is given. Enrichments in r0 compared to r1/r2 with ratios above 1.66 are marked with black brackets, depletions with ratios below 0.66 are marked with grey brackets.

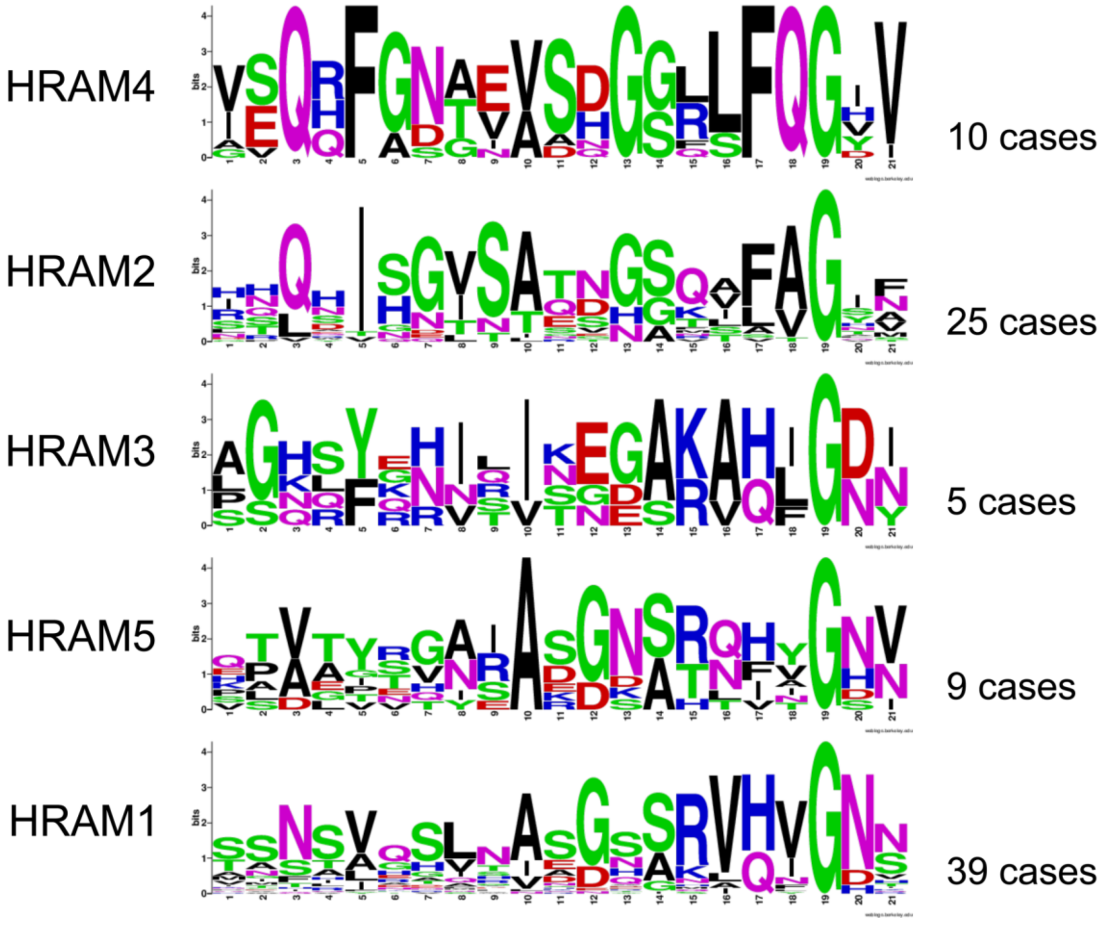

Figure S4. Clustering of HET-s-related motifs from NLR proteins.

Sequence logos defining subfamilies of HET-s-related motifs from fungal NLR proteins obtained at PAM130 are given. The logos were generated for five most numerous groups (including 88 out of 95 motifs).
